# Survival Rate of the Neotropical Stingless Bees *Nannotrigona perilampoides* and *Frieseomelitta nigra* after Exposure to Five Selected Insecticides, under Controlled Conditions

**DOI:** 10.3390/insects13100961

**Published:** 2022-10-20

**Authors:** Cristian Góngora-Gamboa, Esaú Ruiz-Sánchez, Horacio S. Ballina-Gómez, Alejandra González-Moreno, Roberto Zamora-Bustillos

**Affiliations:** División de Estudios de Posgrado e Investigación, TecNM/Instituto Tecnológico de Conkal, Av. Tecnológico s/n, Conkal 97345, Mexico

**Keywords:** stingless bees, neonicotinoid insecticides, diamide insecticide, butenolide insecticide, cyclic keto-enol insecticide, nAChR-acting insecticides

## Abstract

**Simple Summary:**

In the neotropics, stingless bees co-exist with plant pests in agroecosystems. The use of chemical insecticides used to control sap-sucking insects may pose a risk to the communities of stingless bees. To gain insight into the potential risk of insecticides commonly used for farmers in horticultural crops, we evaluated under laboratory conditions the effects of oral exposure to five insecticides on the survival of two species of stingless bees, *Nannotrigona* *perilampoides* and *Frieseomelitta nigra*. The results showed that some insecticides have a significant negative impact on the survival of stingless bees under laboratory conditions. These results suggest that stingless bees may be negatively affected when foraging flowers of crops that have been treated with insecticides.

**Abstract:**

Insecticides used in agricultural pest management pose survival risks to the stingless bees that forage on crops in tropical and subtropical regions. In the present study, we evaluated, under laboratory conditions, the acute oral toxicity of five selected insecticides (dinotefuran, imidacloprid, flupyradifurone, spirotetramat, and cyantraniliprole) to two species of neotropical stingless bees: *Nannotrigona perilampoides* and *Frieseomelitta nigra*. At field recommended doses, dinotefuran, imidacloprid, and flupyradifurone caused the highest mortality in both bee species. These insecticides also caused the largest decrease in the survival rate when exposed to a 10-fold dilution of the field recommended doses. Notably, dinotefuran exerted a high effect even at 100-fold dilution (100% mortality). In contrast, cyantraniliprole had a low effect and spirotetramat was virtually nontoxic. These results suggest that some insecticides used to control sap-sucking insects may have a significant negative impact on the communities of stingless bees.

## 1. Introduction

Stingless bees (Hymenoptera: Meliponini) are important pollinators to wild and cultivated plants [1]. In some regions of southeastern Mexico, stingless bees are particularly associated with economically important local species crops. For example, in the Yucatán Peninsula, Mexico, a region with a high diversity and abundance of stingless bees, *Nannotrigona perilampoides* and *Frieseomelitta nigra* are efficient pollinators of peppers, tomatoes, and avocado [2,3,4], and of important native plants such as annatto, also known as “*achiote*” (*Bixa orellana*) and *Jatropha curcas*, used for the production of biofuel [5,6]. Furthermore, the stingless bees *Melipona beecheii* pollinate *achiote* [6] and *Scaptotrigona pectoralis*, and *Trigona fulviventris* pollinate chili peppers (*Capsicum chinense*) [7], which shows that stingless bees play an important role in maintaining the diversity of the flora.

In the last couple of decades, a reduction in the richness and abundance of native stingless bees has been experienced, which has been associated with various factors, such as the anthropogenic disturbance and high dominance of the Africanized honeybee [8], the loss of forests [9,10], and the presence of insecticides in the environment [11].

In intensively managed agroecosystems, stingless bees are exposed to a variety of chemical insecticides when foraging flowers [12,13]. In this sense, to control highly damaging sap-sucking insects, farmers of the region have made intense use of four groups of insecticides: neonicotinoids (imidacloprid, thiamethoxam, and dinotefuran), butenolides (flupyradifurone), cyclic keto-enols (spirotetramat), and diamides (cyantraniliprole) [13,14,15]. The use of these novel groups of insecticides has been questioned due to their negative impact upon and threat to the bee community [16].

To gain insight into the potential risks of insecticide use in horticultural crops, we evaluated under laboratory conditions the effects of oral exposure to dinotefuran, imidacloprid, flupyradifurone, spirotetramat, and cyantraniliprole to the survival of two species of stingless bees, *Nannotrigona perilampoides* and *Frieseomelitta nigra*.

## 2. Materials and Methods

### 2.1. Insects and Insecticides

The research was carried out at the Tecnológico Nacional de México, Campus Conkal, at Conkal, Yucatán, Mexico. The colonies of *N. perilampoides* and *F. nigra* were maintained in wooden hives in the field (five hives per bee species) and allowed to forage naturally. The vegetation surrounding the hives included *Piscidia piscipula*, *Leucaena leucocephala*, *Tithonia diversifolia*, and *Parthenium hysterophorus*. In the feeding zone of the stingless bees, the absence of agricultural crops under the application of agrochemicals was verified. Additionally, twice a week, 10 mL of a 50% sucrose solution (1 g of sucrose: 1 mL of distilled water) was provided to each hive as a diet source.

The commercial formulations of the insecticides were purchased from local agrochemical suppliers. All the insecticides evaluated (Table 1) are recommended to control sap-sucking insects (Aleyrodidae and Aphididae) in horticultural crops. The concentrations of the insecticides used for the evaluation were selected based on the application rate recommended by the manufacturer for the whitefly (*Bemisia tabaci*). From these field recommended doses, 10-fold and 100-fold dilutions were prepared for the experiment of survival analysis.

### 2.2. Oral Toxicity Bioassay

Groups of 10 foragers were collected from the entrance of the hives of the stingless bees. Each group, obtained from a different hive, was placed in a plastic bottle (500 mL) with filter paper on the bottom and a 20 cm^2^ section of organza mesh was fixed to the side of the bottle to facilitate the air exchange. In the middle part of the bottle (the organza mesh section) we placed a microtube (1.5 mL) for food supply, which consisted of sucrose solution (sucrose/water 1:1) ad libitum. To minimize the stress caused by confinement, prior to the bioassays the bees remained in adaptation for approximately 12 h (one night and part of the next morning) at room conditions, 25 °C and 70% R.H. 

The oral acute toxicity test was conducted in adult foragers as described by Botina et al. [17]. Prior to insecticide exposure, bees were starved for 2 h. Following this, 1 mL of insecticide-contaminated diet was placed in the microtube placed at the middle part of the bottle. Insecticide-free diet was used as control. The mortality was recorded after 2, 4, and 24 h of offering the diet. Individuals were considered dead if they did not react when stimulated with a fine hairbrush [18,19]. The experiment was set in a completely randomized design with five replicates per insecticide, each experimental unit consisting of 10 foragers confined in a 500 mL plastic bottle.

### 2.3. Statistical Analysis

Analysis of variance (ANOVA) was used to compare the mortality caused by the field recommended doses of the insecticides over 24 h. Comparison of means was carried out with Tukey post hoc test at *p* < 0.05. The normality and homoscedasticity of the data were confirmed prior to their analysis. Survival curves were obtained with the Kaplan–Meier estimators, which were generated from the percentage of mortality of bees over 2, 4, and 24 h evaluation periods with 10-fold and 100-fold dilution of the insecticides. The estimated survival functions were compared with a nonparametric Log-Rank test (Holm–Sidak method). All the statistical analyses were performed using the SigmaPlot 11.0 software (Systat Software, San Jose, CA, USA).

## 3. Results

### 3.1. Mortality

**Figure 1:**
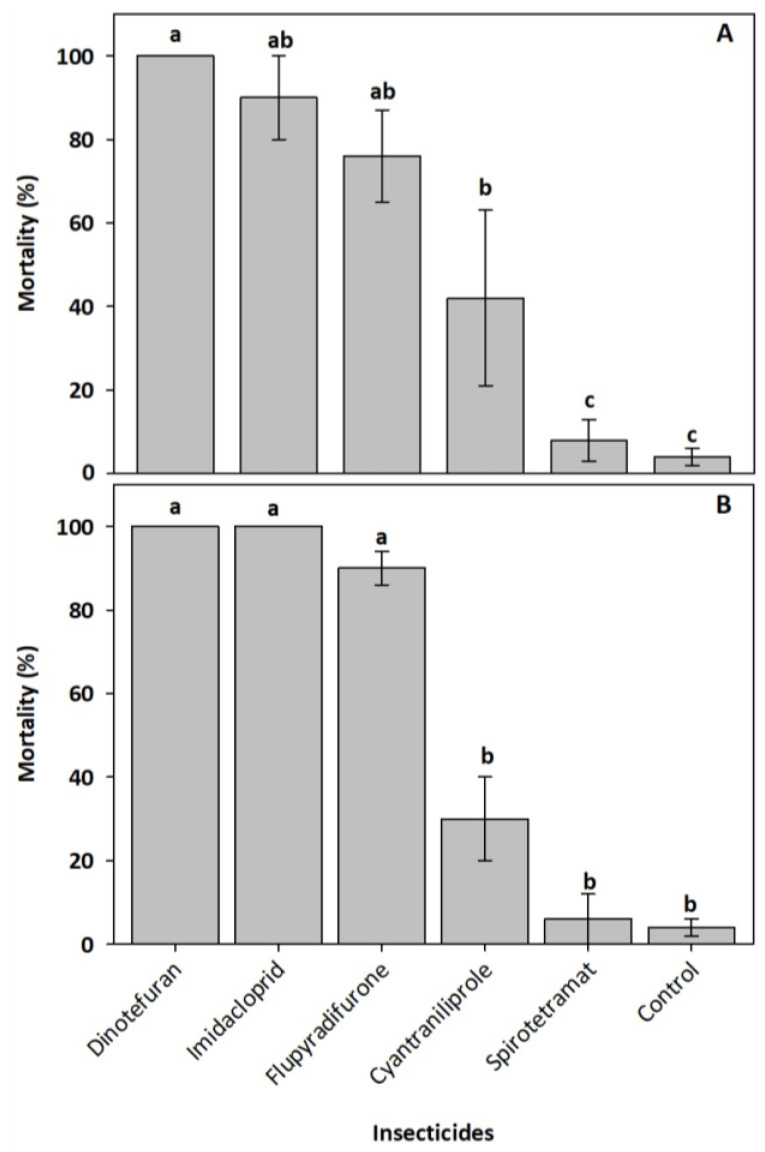
Mortality percentage (± standard error) of the stingless bees *Nannotrigona perilampoides* (**A**) and *Frieseomelitta nigra* (**B**), orally exposed for 24 h to the insecticides at field recommended doses, dinotefuran (600 mg a.i. L^−1^), imidacloprid (700 mg a.i. L^−1^), flupyradifurone (680 mg a.i. L^−1^), spirotetramat (300 mg a.i. L^−1^), and cyantraniliprole (200 mg a.i. L^−1^). Bars with different letters are statically different (Tukey test, n = 5, *p* < 0.05).

Oral exposure for 24 h to the field recommended doses of insecticides caused significant differences in mortality in *N. perilampoides* (*F* = 14.03, df = 5, *p* < 0.0001) and *F. nigra* (*F* = 76.06, df = 5, *p* < 0.0001). In both bee species, dinotefuran, imidacloprid, and flupyradifurone were the most toxic. Bee mortality ranged from 90–100% when exposed to dinotefuran and imidacloprid, and from 75–90% when exposed to flupyradifurone (Figure 1). Mortality caused by cyantraniliprole was lower (30–45%). Spirotetramat was not toxic to either bee species (Figure 1).

### 3.2. Survival Analysis

**Figure 2:**
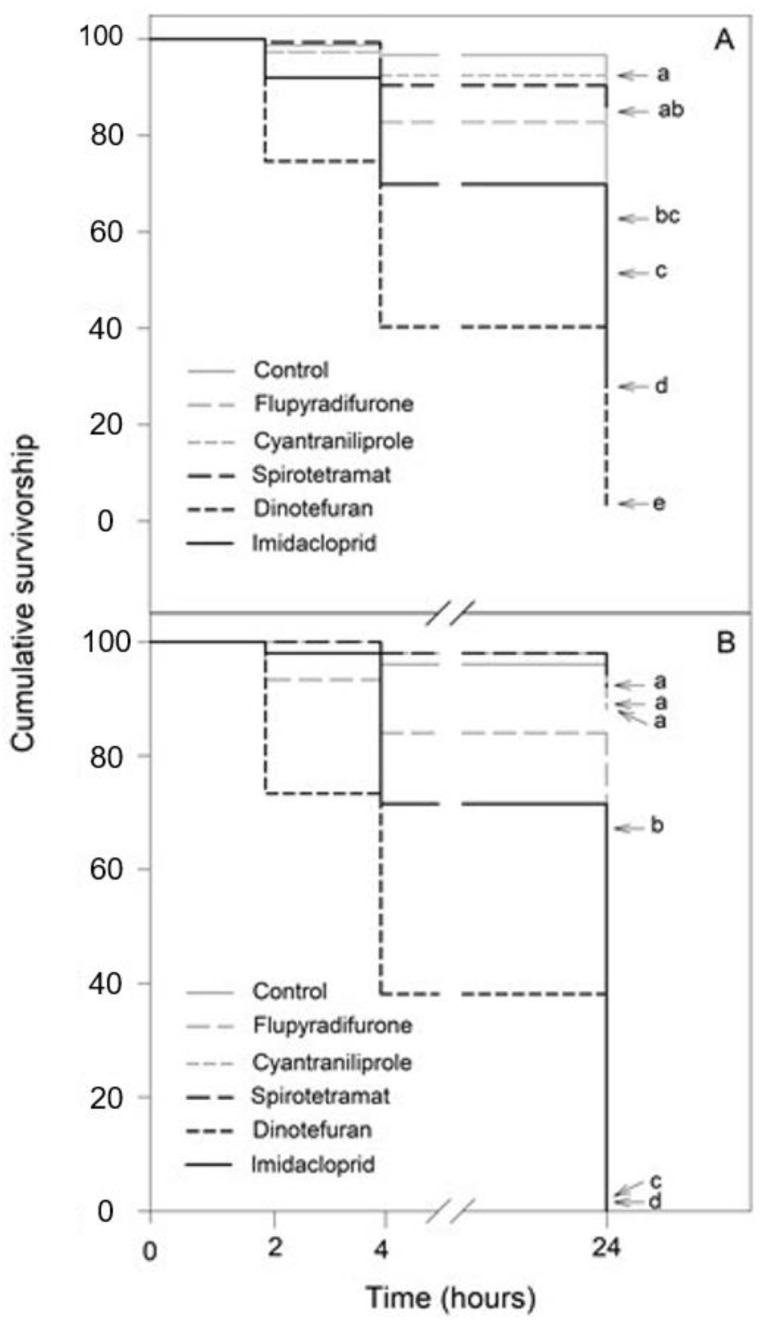
Kaplan–Meier survivorship curves of stingless bees orally exposed to 10-fold diluted commercial formulation of the selected insecticides: dinotefuran, 60 mg L^−1^; imidacloprid, 70 mg L^−1^; flupyradifurone, 68 mg L^−1^; spirotetramat, 30 mg L^−1^; and cyantraniliprole, 20 mg L^−1^. The figure displays *Nannotrigona perilampoides* (**A**) and *Frieseomelitta nigra* (**B**). The estimated survival functions were compared with a nonparametric Log-Rank test (Holm–Sidak method). Significant differences (*p* < 0.05) among insecticide treatments are indicated by different letters.

Exposure to the insecticides, in a 10-fold dilution and 100-fold dilution, resulted in significantly distinctive effects in both bee species (Tables S1–S4): *N. perilampoides* (χ^2^ = 300.8, df = 5, *p* < 0.001; Figure 2A) and *F. nigra* (χ^2^ = 432.9, df = 5, *p* < 0.001; Figure 2B), depending on the agrochemical used. Overall, in both species, dinotefuran, and imidacloprid produced the largest decrease in the survival rate, followed by flupyradifurone. Cyantraniliprole had a slight, but significant effect, and spirotetramat showed no toxicity (Figure 2).

**Figure 3:**
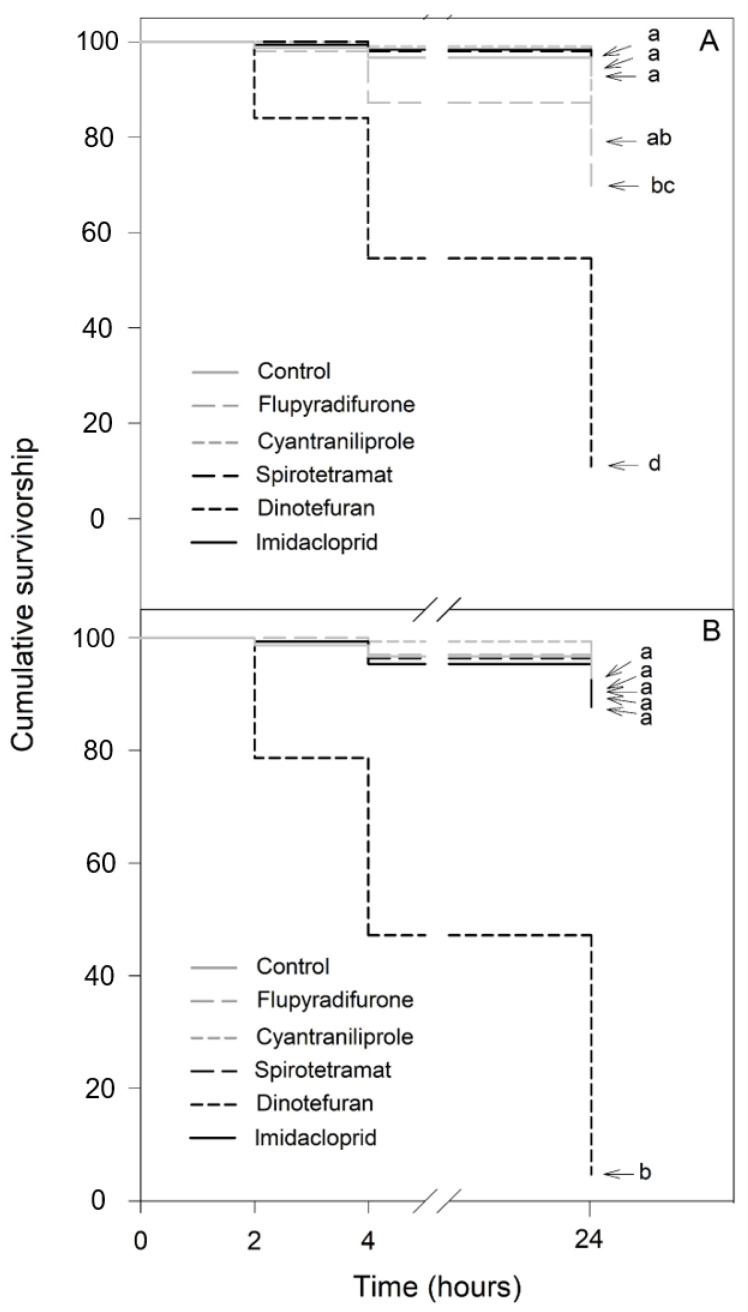
Kaplan–Meier survivorship curves of stingless bees orally exposed to 100-fold diluted commercial formulation of the selected insecticides: dinotefuran, 6 mg L^−1^; imidacloprid, 7 mg L^−1^; flupyradifurone, 6.8 mg L^−1^; spirotetramat, 3 mg L^−1^; and cyantraniliprole, 2 mg L^−1^. The figure displays *Nannotrigona perilampoides* (**A**) and *Frieseomelitta nigra* (**B**). The estimated survival functions were compared with a nonparametric Log-Rank test (Holm–Sidak method). Significant differences (*p* < 0.05) among insecticide treatments are indicated by different letters.

For the 100-fold dilution of the recommended application rate, significant differences in the effects of the insecticides were observed depending on the bee species (Figure 3). In *N. perilampoides* (χ^2^ = 322.1, df = 5, *p* < 0.001), flupyradifurone and dinotefuran caused significant decreases in the survival rates: the highest effect was produced by dinotefuran (90% mortality), while cyantraniliprole, spirotetramat, and imidacloprid had no effect (Figure 3A). On the other hand, in *F. nigra* (χ^2^ = 466.6, df = 5, *p* < 0.001) dinotefuran produced a complete depletion of the survival rate (100% mortality). The other insecticides (flupyradifurone, cyantraniliprole, spirotetramat, and imidacloprid) had no effect (Figure 3B).

## 4. Discussion

In the present study, we evaluated the effects of oral exposure to selected insecticides on the survival of two species of neotropical stingless bees, *N. perilampoides* and *F. nigra*, under laboratory conditions. It is worth considering that oral exposure may negatively impact not only the survival of the directly exposed bees, but also the bees in the colonies, given that foragers, who collect contaminated pollen or nectar, carry them to the hives [20,21,22].

Here, we observed that at field recommended doses, dinotefuran, imidacloprid, and flupyradifurone (nAChR-acting insecticides) exerted the strongest lethal effects in both bee species. For dinotefuran and imidacloprid, a dramatic decrease in the survival rate (approximately 50–60% mortality) was observed as quickly as 4 h after exposure to the 10-fold dilution of the field recommended doses. Moreover, we observed that even at 100-fold dilution, dinotefuran was able to dramatically deplete the bee survival rate (100% mortality). In this sense, previous studies have shown that nAChR-acting insecticides are highly toxic to various species of bees [23,24,25], and their lethal effects at realistic concentrations are observed as quickly as 1 h after insect exposure [26]. In the present work, we also found that flupyradifurone caused a significant decrease in the survival rate of *N. perilampoides* and *F. nigra* when used at the 10-fold dilution. Flupyradifurone is considered a highly toxic insecticide to the European bee (*Apis mellifera*), which may indicate that this insecticide would be even more toxic to stingless bees, given their high susceptibility to insecticides [27,28]. 

The anthranilic diamide, cyantraniliprole, showed low to moderate toxicity to both species of bees at the 10-fold dilution, but had no effect at all at the 100-fold dilution. To our knowledge, this insecticide has not been previously evaluated on stingless bees. However, Tomé et al. [29] found that chlorantraniliprole (3 mg L^−1^), another anthranilic diamide compound, affects the flight activity of *Partamona helleri* and *Scaptotrigona xanthotrica*. In *Apis mellifera*, compounds from this group of insecticides have moderate effects when applied topically [30]. In agriculture, cyantraniliprole is recommended to control phytophagous species of the families Thripidae, Liviidae, Aphididae, Aleyrodidae, Noctuidae, Plutellidae, and Gelechiidae [14,31,32,33]. In contrast to nAChR-acting insecticides, anthranilic diamide insecticides act on ryanodine receptors (RyR), which are responsible for intracellular calcium regulation within the sarcoplasmic reticulum of insect muscle cells [34], but with differential effects among insect orders and even among species [35,36]. This difference in susceptibility has been attributed to the binding affinity of the insecticide molecule to the target site [37]. It is reasonable to infer that the low to moderate toxicity of cyantraniliprole to the stingless bees, shown in the present work, may be in part due to this pharmacological characteristic of the anthranilic diamide insecticides. 

The cyclic keto-enol, spirotetramat, had no acute oral effects on both species of stingless bees. Spirotetramat has been recommended to control both immature and adult stages of phytophagous mites [38,39], and immature Hemiptera of the families Aleyrodidae, Aphididae, and Triozidae [40,41]. Spirotetramat is particularly effective against the juvenile stages of pest insects; the compound also reduces fecundity and fertility in adults [40]. Owing to its mode of action (the disruption of lipid biosynthesis by inhibiting the Acetyl-CoA-carboxylase), spirotetramat has low toxicity to adult insects [14,42], which would explain why this insecticide was not toxic to either species of stingless bee. This outcome is in agreement with other studies that showed the nontoxic effects of spirotetramat on adults of *A. mellifera* [43,44], and adults of *Apis cerana*, *Apis florea*, and *Trigona iridipennis* [44]. In this work, we did not evaluate the effects of spirotetramat on the immature stages of the stingless bees, thus we could not rule out the potential effects of this compound when ingested by larvae in contaminated nectar/pollen carried by foragers to the hives.

## 5. Conclusions

The acute oral exposure of *N. perilampoides* and *F. nigra* to the nAChR-acting insecticides dinotefuran, imidacloprid, and flupyradifurone, negatively impacted their survival rate. Even at a low concentration (100-fold of the recommended application rate), dinotefuran produced a dramatic impact on bee survival, whereas insecticides that act on ryanodine receptors (cyantraniliprole) or inhibit lipid biosynthesis (spirotetramat) caused moderate and nontoxic acute effects, respectively. The results show that the intensive use of the nAChR-acting insecticides (dinotefuran, imidacloprid and flupyradifurone) to control sap-sucking insects in horticultural crops poses a threat to the communities of neotropical stingless bees that forage in the area. It is important to evaluate the impact of these insecticides on the expression of genes involved in metabolic pathways as well as to evaluate the epigenetic changes after long term exposure, in order to gain insight on the effects of these insecticides at physiological and molecular levels in stingless bees. 

## Figures and Tables

**Table 1:** Details of the insecticides evaluated in the present experiment.

Insecticide (Chemical Group)	Concentration (Formulation) ^a^	Evaluated Concentrations (mg L^−1^) *	Trade Name (Manufacturer)	Mode of Action
Dinotefuran (Neonicotinoid)	200 g/kg (SG)	600, 60, 6	Venom 20 SG (Valent)	Agonist of Nicotinic Acetylcholine receptor (nAChR)
Imidacloprid (Neonicotinoid)	350 g a.i. L^−1^ (SC)	700, 70, 7	Confial (Quimica Sagal)	Agonist of Nicotinic Acetylcholine receptor (nAChR)
Flupyradifurone (Butenolide)	200 g a.i. L^−1^ (SL)	680, 68, 6.8	Sivanto Prime (Bayer Crop Science)	Agonist of Nicotinic Acetylcholine receptor (nAChR)
Spirotetramat (Cyclic keto-enol)	150 g a.i. L^−1^ (OD)	300, 30, 3	Movento 150 OD (Bayer Crop Science)	Inhibitor of acetyl CoA carboxilase
Cyantraniliprole (Anthranilic diamide)	200 g L^−1^ (SC)	200, 20, 2	Benevia (FMC)	Activator of Ryanodine Receptor (RyR)

* The highest concentrations of the insecticides used for the evaluation were based on the field recommended doses for the whitefly (*Bemisia tabaci*) and their 10-fold and 100-fold dilution. ^a^ Concentration and (formulation). SG: soluble granules; SC: suspension concentrate; SL: soluble concentrate; OD: oil dispersion.

## Data Availability

The data are available upon request from the corresponding authors esau.rs@conkal.tecnm.mx and roberto.zb@conkal.tecnm.mx.

## Supplementary Materials

The following are available online at https://www.mdpi.com/article/10.3390/insects13100961/s1, Tables S1–S4. Evaluation of 10–100-fold dilution of the recommended application rate.

## References

[1] Meléndez-Ramírez V., Ayala R., Delfín-González H., Vit P., Pedro S.R.M., Roubik D.W. (2018). Crop pollination by stingless bees. Pot-Pollen in Stingless Bee Melittology.

[2] Cauich O., Quezada-Euán J.J.G., Macias-Macias J.O., Reyes-Oregel V., Medina-Peralta S., Parra-Tabla V. (2004). Behavior and pollination efficiency of *Nannotrigona perilampoides* (Hymenoptera: Meliponini) on greenhouse tomatoes (*Lycopersicon esculentum*) in subtropical Mexico. J. Econ. Entomol..

[3] Can-Alonzo C., Quezada-Euán J.J.G., Ancona-Xiu P., Moo-Valle H., Valdovinos-Nuñez G.R., Medina-Peralta S. (2005). Pollination of ‘criollo’ avocados (*Persea americana*) and the behaviour of associated bees in subtropical Mexico. J. Apic. Res..

[4] Cauich O., Quezada-Euán J.J.G., Meléndez-Ramírez V., Valdovinos-Nuñez G.R., Moo-Valle H. (2006). Pollination of habanero pepper (*Capsicum chinense*) and production in enclosures using the stingless bee *Nannotrigona perilampoides*. J. Apic. Res..

[5] Romero M.J., Quezada-Euán J.J.G. (2013). Pollinators in biofuel agricultural systems: The diversity and performance of bees (Hymenoptera: Apoidea) on *Jatropha curcas* in Mexico. Apidologie.

[6] Caro A., Moo-Valle H., Alfaro R., Quezada-Euán J.J.G. (2016). Pollination services of Africanized honey bees and native *Melipona beecheii* to buzz-pollinated annatto (*Bixa orellana* L.) in the neotropics. Agric. For. Entomol..

[7] Landaverde-González P., Quezada-Euán J.J.G., Theodorou P., Murray T.E., Husemann M., Ayala R., Moo-Valle H., Vandame R., Paxton J.R. (2017). Sweat bees on hot chillies: Provision of pollination services by native bees in traditional slash-and-burn agriculture in the Yucatán Peninsula of tropical Mexico. J. Appl. Ecol..

[8] Cairns C.E., Villanueva-Gutiérrez R., Koptur S., Bray B.D. (2005). Bee populations, forest disturbance, and africanization in Mexico. Biotropica.

[9] Brosi B.J. (2009). The complex responses of social stingless bees (Apidae: Meliponini) to tropical deforestation. For. Ecol. Manag..

[10] Mayes D.M., Bhatta C.P., Shi D., Brown J.C., Smith D.R. (2019). Body size influences stingless bee (Hymenoptera: Apidae) communities across a range of deforestation levels in Rondônia, Brazil. J. Insect Sci..

[11] Main A.R., Webb E.B., Goyne K.W., Mengel D. (2020). Reduced species richness of native bees in field margins associated with neonicotinoid concentrations in non-target soils. Agric. Ecosyst. Environ..

[12] Macias-Macias O., Chuc P., Ancona-Xiu P., Cauich O., Quezada-Euán J.J.G. (2009). Contribution of native bees and Africanized honeybees (Hymenoptera: Apoidea) to Solanaceae crop pollination in tropical México. J. Appl. Entomol..

[13] Montejo-Canul E., Aguiñaga-Bravo A., Ruiz-Sánchez E., Ballina-Gómez H., González-Moreno A., Latournerie-Moreno L., Martín-Mex R., Garruña-Hernández R. (2019). Effects of the inclusion of biorational insecticides for pest management on phytophagous insects, fruit yield, and bee abundance in tomato and tomatillo. Arch. Phytopathol. Pflanzenschutz.

[14] Chen J.-C., Wang Z.-H., Cao L.-J., Gong Y.-J., Hoffmann A.A., Wei S.-J. (2018). Toxicity of seven insecticides to different developmental stages of the whitefly *Bemisia tabaci* MED (Hemiptera: Aleyrodidae) in multiple field populations of China. Ecotoxicology.

[15] Monroy-Reyes B., Carrillo-Gutiérrez T., Beas-Zarate C., Posos-Ponce P., Castro-Rodriguez M., Enciso-Cabral J.G., Flores-Galano G. (2019). Evaluación de efectividad biológica del insecticida Benevia 10 OD (ciantraniliprol) para el control de *Frankliniella occidentalis* pergande, (Thysanoptera: Thripidae) en aguacate. Entomología Mexicana.

[16] Siviter H., Muth F. (2020). Do novel insecticides pose a threat to beneficial insects?. Proc. R. Soc. B.

[17] Botina L.L., Bernardes R.C., Barbosa W.F., Lima M.A.P., Guedes R.N.C., Martins G.F. (2020). Toxicological assessments of agrochemical effects on stingless bees (Apidae, Meliponini). MethodsX.

[18] Lourenço C.T., Carvalho S.M., Malaspina O., Nocelli R.C.F. (2012). Oral toxicity of fipronil insecticide against the stingless bee *Melipona scutellaris* (Latreille, 1811). Bull. Environ. Contam. Toxicol..

[19] Jacob C.R.O., Malaquias J.B., Zanardi O.Z., Silva C.A.S., Jacob J.F.O., Yamamoto P.T. (2019). Oral acute toxicity and impact of neonicotinoids on *Apis mellifera* L. and *Scaptotrigona postica* Latreille (Hymenoptera: Apidae). Ecotoxicology.

[20] Sanchez-Bayo F., Goka K. (2014). Pesticide residues and bees–a risk assessment. PLoS ONE.

[21] Lima M.A.P., Martins G.F., Oliveira E.E., Guedes R.N.C. (2016). Agrochemical-induced stress in stingless bees: Peculiarities, underlying basis, and challenges. J. Comp. Physiol. A.

[22] Cham K.O., Nocelli R.C.F., Borges L.O., Viana-Silva F.E.C., Tonelli C.A.M., Menezes O.M.C., Rosa-Fontana A.S., Blochtein B., Freitas B.M., Pires C.S.S. (2018). Pesticide exposure assessment paradigm for stingless bees. Environ. Entomol..

[23] Cresswell J.E. (2011). A meta-analysis of experiments testing the effects of a neonicotinoid insecticide (imidacloprid) on honey bees. Ecotoxicology.

[24] Goulson D. (2013). An overview of the environmental risks posed by neonicotinoid insecticides. J. Appl. Ecol..

[25] Arena M., Sgolastra F. (2014). A meta-analysis comparing the sensitivity of bees to pesticides. Ecotoxicology.

[26] Tosi S., Nieh J.C. (2019). Lethal and sublethal synergistic effects of a new systemic pesticide, flupyradifurone (Sivanto^®^), on honeybees. Proc. R. Soc B.

[27] Nauen R., Jeschke P., Velten R., Beck M.E., Ebbinghaus-Kintscher U., Thielert W., Wölfel K., Hass M., Kunz K., Raupach G. (2015). Flupyradifurone: A brief profile of a new butenolide insecticide. Pest Manag. Sci..

[28] Hesselbach H., Scheiner R. (2018). Effects of the novel pesticide flupyradifurone (Sivanto) on honeybee taste and cognition. Sci. Rep..

[29] Tomé H.V.V., Barbosa W.F., Corrêa A.S., Gontijo L.M., Martins G.F., Guedes R.N.C. (2015). Reduced-risk insecticides in neotropical stingless bee species: Impact on survival and activity. Ann. Appl. Biol..

[30] Kadala A., Charreton M., Charnet P., Collet C. (2019). Honey bees long-lasting locomotor deficits after exposure to the diamide chlorantraniliprole are accompanied by brain and muscular calcium channels alterations. Sci. Rep..

[31] Zhang Z., Xu C., Ding J., Zhao Y., Lin J., Liu F., Mu W. (2019). Cyantraniliprole seed treatment efficiency against *Agrotis ipsilon* (Lepidoptera: Noctuidae) and residue concentrations in corn plants and soil. Pest Manag. Sci..

[32] Li Y., Dou Y., An J., Tu X., Lv H., Pan W., Dang Z., Gao Z. (2020). Temperature-dependent variations in toxicity of diamide insecticides against three lepidopteran insects. Ecotoxicology.

[33] Renkema J.M., Krey K., Devkota S., Liburd O.E., Funderburk J. (2020). Efficacy of insecticides for season-long control of thrips (Thysanoptera: Thripidae) in winter strawberries in Florida. Crop Prot..

[34] Selby T.P., Lahm G.P., Stevenson T.M., Hughes K.A., Cordova D., Annan I.B., Barry J.D., Benner E.A., Currie M.J., Pahutski T.F. (2013). Discovery of cyantraniliprole, a potent and selective anthranilic diamide ryanodine receptor activator with cross-spectrum insecticidal activity. Bioorg. Med. Chem. Lett..

[35] Isaacs A.K., Qi S., Sarpong R., Casida J.E. (2012). Insect ryanodine receptor: Distinct but coupled insecticide binding sites for N-C(3)H(3) chlorantraniliprole, flubendiamide, and (3)H ryanodine. Chem. Res. Toxicol..

[36] Dinter A., Samel A. Cyantraniliprole: Pollinator profile of the novel insecticides under laboratory, semi-field and field conditions. Proceedings of the Hazards of Pesticides to Bees–12th International Symposium of the ICP-Bee Protection Group.

[37] Qi S., Casida J.E. (2013). Species differences in chlorantraniliprole and flubendiamide insecticide binding sites in the ryanodine receptor. Pestic. Biochem. Phys..

[38] Lümmen P., Khajehali J., Luther K., Van Leeuwen T. (2014). The cyclic keto-enol insecticide spirotetramat inhibits insect and spider mite acetyl-CoA carboxylases by interfering with the carboxyltransferase partial reaction. Insect Biochem. Mol. Biol..

[39] Popov S.Y., Alyokhin A. (2019). Gender-Specific acaricidal properties and sexual transmission of spirotetramat in two-spotted spider mite (Tetranychidae: Acariformes). J. Econ. Entomol..

[40] Nauen R., Reckmann U., Thomzik J., Thielert W. (2008). Biological profile of spirotetramat (Movento^®^)–a new two-way systemic (ambimobile) insecticide against sucking pest species. Bayer Cropsci. J..

[41] Brück E., Elbert A., Fischer R., Krueger S., Kühnhold J., Klueken A.M., Nauen R., Niebes J.F., Reckmann U., Schnorbach H.J. (2009). Movento^®^, an innovative ambimobile insecticide for sucking insect pest control in agriculture: Biological profile and field performance. Crop Prot..

[42] Marčić D., Perić P., Petronijević S., Prijović M., Drobnjaković T. (2011). Cyclic ketoenols: Acaricides and insecticides with a novel mode of action. Pestic. I Fitomed..

[43] Maus C. (2008). Ecotoxicological profile of the insecticide spirotetramat. Bayer Cropsci. J..

[44] Vinothkumar B., Kumaran N., Boomathi N., Saravanan P.A., Kuttalam S. (2010). Toxicity of spirotetramat 150 OD to honeybees. Madras Agric. J..

